# Knowledge, attitudes, and practices about homelessness and willingness-to-pay for housing-first across 8 European countries: a survey protocol

**DOI:** 10.1186/s13690-018-0317-x

**Published:** 2018-11-28

**Authors:** J. M. Petit, S. Loubiere, M. J. Vargas-Moniz, A. Tinland, F. Spinnewijn, R. M. Greenwood, M. Santinello, J. R. Wolf, A. Bokszczanin, R. Bernad, H. Kallmen, J. Ornelas, P. Auquier, HOME-EU consortium study group, Freek Spinnewijn, Pascal Auquier, Junie M. Petit, Sandrine Loubière, Aurelie Tinland, Ronni M. Greenwood, Rachel M. Manning, Branagh O’Shaughnessy, Francesca Disperati, Marta Gaboardi, Michela Lenzi, Massimo Santinello, Alessio Vieno, Yvonne Peters, Tessa van Loenen, Liselotte Raben, Judith R. Wolf, Anna Bokszczanin, Barbara Zmaczynska–Witek, Skałacka Katarzyna, Inês Almas, Teresa Duarte, Paulo Martins, Tiago Cruz, Carla Pereira, Rita P. Marques, Américo Nave, Maria J. Vargas-Moniz, Maria F. Jorge-Monteiro, José Ornelas, Roberto Bernad, Borja Rivero, Ulla Beijer, Mats Blid, Hakan Kallmen

**Affiliations:** 1 Aix-Marseille Univ, School of medicine – La Timone Medical Campus, EA 3279 CEReSS – Health Service Research and Quality of Life Center, 27 Boulevard Jean Moulin, 13385 Marseille, France; 2 Department of Research and Innovation, Support Unit for clinical research and economic evaluation, Assistance Publique – Hôpitaux de Marseille, 27 Boulevard Jean Moulin, 13385 Marseille, France; 3 Applied Psychology Research Center: Capabilities and Inclusion (APPsyCI), ISPA-Instituto Universitário, Rua Jardim do Tabaco, 34, 1149-041 Lisbon, Portugal; 4 FEANTSA, European Federation of National Organisations Working with the Homeless, 194, Chaussée de Louvain, 1210 Brussels, Belgium; 5 Department of Psychology, University of Limerick, Limerick, V94 T9PX Ireland; 6 Department of Developmental and Social Psychology, University of Padova, Via Venezia, 8 - 35131, Padova, Italy; 7 Impuls - Netherlands Center for Social Care Research, Radboud Institute for Health Sciences, Geert Grooteplein 27, 6525 EZ Nijmegen, The Netherlands; 8 Institute of Psychology, Opole University, Pl. Staszica 1, 45-052 Opole, Poland; 9 Rais Fundación, C/ Ardemans 42, 28028 Madrid, Spain; 10 STAD, Stockholm Center for Psychiatry Research and Education, Karolinska Institutet, Norra Stationsgatan 69, 113 64 Stockholm, Sweden

**Keywords:** Housing First, Homelessness, Contingent valuation, Willingness-to-pay (WTP), Knowledge, attitudes, practices (KAP), Europe, Bidding game, Public opinion

## Abstract

**Background:**

Most European countries report rising numbers of people experiencing homelessness. For those with mental disorders, interventions are centered on achieving mental health and drug rehabilitation alongside housing readiness, often to the detriment of access to housing. Notwithstanding, more European countries are investing in a new model, *Housing First (HF),* which postulates immediate access to permanent housing with no initial requirements for treatment. While results of the European HF programs are published on individual-level data, little is known about the opinions of the general population about homelessness and the societal value of the HF model, which can represent barriers to the model’s dissemination. Therefore, we present the protocol of a study designed for the following objectives: 1) to explore the knowledge, attitudes, and practices (KAP) about homelessness within the general population of 8 European countries, 2) to assess the valuation of the HF model by European citizens, and 3) to estimate the lifetime prevalence of homelessness in the targeted countries.

**Methods:**

A telephone survey was conducted from March to December 2017 among adults selected from opt-in panels from France, Ireland, Italy, the Netherlands, Portugal, Spain, Poland, and Sweden. A total sample of 5600 interviews was expected, with 700 per country. The interviews included three sections: first, the KAP about homelessness; second, the valuation of the HF model by measuring a respondent’s willingness-to-pay (WTP) through the contingent valuation method; and third, an assessment of the lifetime prevalence of homelessness among the general population. Descriptive analyses and comparisons between countries will be conducted. KAP indicators will be created and their psychometric properties assessed. Determinants of WTP will be assessed through regression models.

**Discussion:**

This survey will highlight Europeans’ views of homelessness, especially their level of tolerance towards homelessness, potential misconceptions and the most important barriers for the implementation of the HF model. Additionally, the results on the valuation of the HF model by citizens could be instrumental for key stakeholders in understanding the level of support from the general population. Ethics approval has been obtained from the Aix-Marseille University Ethics Committee (n° 2016-01-02-01) for this study, which is part of HOME_EU: Reversing Homelessness in Europe H2O20-SC6-REVINEQUAL-2016/GA726997.

## Background

In Europe, updated and reliable estimates of the number of people experiencing homelessness are limited [1]. The latest estimates indicate that four million people were homeless in 2009 [2]. The rise of homelessness in this growing context of inequalities [3] can have severe consequences at the societal and at an individual level. Indeed, society has to bear the costs of homelessness in a number of ways, for example, by providing dedicated services to people experiencing homelessness, as well as health care and social support through mainstream services, and likewise, for the criminal justice system [4–8]. At the individual level, the physical health and mental health of people experiencing homelessness are impacted, with a shorter lifespan and higher rates of infections, heart disease, substance abuse, and psychosis when compared to the general population [5, 9, 10]. Research suggests a higher prevalence of psychosis in people experiencing homelessness, ranging from 3 to 42% compared to 1–2% in the general population [10].

Since the 1980s, the continuum of care model has been in use, in which the functioning level of a homeless person dictates the type of temporary housing he or she should regress or progress to [11]. Interventions within this model have been centered on achieving mental health and/or drug rehabilitation and housing readiness, often to the detriment of access to housing [12, 13]. Studies have shown the limits of such a model in gaining access to independent housing, as people with mental disorders need constant — instead of decreasing — social and healthcare support [11, 14, 15].

The “*Housing-First*” (HF) model was introduced in the USA in 1992, with the potential to effect a shift in the care of people experiencing homelessness. The HF model holds that housing is not a reward but a basic right and is a prerequisite for recovery and community integration [16]. Within the HF model, long-term homeless people presenting psychiatric disorders and/or addiction problems have immediate access to permanent housing, i.e., with no initial requirement for treatment, and have support from a multidisciplinary team [17, 18]. The first results of most HF programs reported from Europe are consistent with those initially undertaken in the United States and Canada; that is, the approach resulted in higher residential stability, better health outcomes, and lower residential and health care costs compared to the continuum of care model [19–21].

So far, most research has focused on outcomes at the individual-level by using results from various HF programs. However, little research has been performed on the views and attitudes of the general population regarding homelessness and programs targeting its reduction in Europe.

The few existing studies of public opinion about homelessness provide concurring results. In particular, a survey among the general population (*n* = 240) of the United States showed that a majority (65.6%) holds society responsible for homelessness and even more (96.9%) support the idea that the government, as opposed to charities, should procure solutions to homelessness [22]. This study also suggested gender as a predictor of attitude, with women more likely to consider homelessness as a worrisome issue gaining momentum and willing to support an increase in federal spending for homelessness and to favor work-oriented interventions as a means of reducing homelessness. Additionally, Tompsett and colleagues linked a higher level of education with seeing homelessness as the result of personal flaws [23]. Only one study conducted in Europe (Germany, Italy, the United Kingdom and Belgium) and in the USA has focused on the opinion of the general population [24]. In this study, small samples were obtained (no more than 323 for Europe and 435 for the USA) and data collection was spread over 5 years. Nonetheless, differences in attitudes about homelessness across countries were found; for example, Italians were more likely to consider economic factors as the primary cause of homelessness when compared to the other countries. Additionally, differences were found in the lifetime prevalence of literal homelessness within the general population, with the highest rates in the UK and in the USA.

In light of the rising numbers of people experiencing homelessness across Europe and of the development of programs aiming to reduce homelessness, key stakeholders would benefit from an updated evaluation of public opinion to understand what drives public support for policies tackling homelessness. Indeed, previous research has shown that public opinion does have a bearing on policy making [25]. Furthermore, as more European countries are investing in Housing First programs, key stakeholders would benefit from a study of its valuation by the general population. Such research aiming at adequately documenting citizens’ willingness-to-pay for healthcare programs have been increasingly used [26, 27] and have the potential of yielding useful information for publicly funded programs in terms of public support, especially in a context of limited resources. To the best of our knowledge, no research has been conducted to explore the preferences of Europeans for the HF model by using the contingent valuation method — a survey-based technique used to elicit willingness-to-pay values [28].

Therefore, we designed a study of which the primary aim is to explore the knowledge, attitudes, and practices (KAP) about homelessness within the general population of eight European countries, namely, France, Ireland, Italy, the Netherlands, Poland, Portugal, Spain, and Sweden. Its secondary aim is to assess the valuation of the HF model by European citizens and analyze the determinants of this valuation. Finally, this study also provides the opportunity to assess the lifetime prevalence of homelessness in the targeted countries. The objective of this paper is to present the method used to carry out this survey.

## Methods

### Study design and procedure

This study is a quota telephone survey using landlines and mobile phones. Respondents were randomly selected from opt-in panels to be representative of the general population with respect to gender and age. Telephone interviews averaged 30 min in length. All interviews were conducted using computer assisted telephone interviews (CATI) software tailored for telephone surveys. Only bilingual interviewers i.e., those speaking French and the language spoken in one of the other 8 countries, were hired and trained on the survey instrument, the software and neutrality techniques. Calls were placed throughout the day, with most taking place during the evening. All interviews were conducted anonymously. To achieve higher response rates, 15 callbacks were made before discarding a telephone number. As an introduction to the interview, respondents were informed of the purpose of the study, the intended use of the data and assured of anonymity.

Interviews were carried out in the targeted countries from March 2017 to December 2017. These interviews included three modules: the first was on the knowledge, attitudes, and practices (KAP) about homelessness; the second covered the contingent valuation (CV) method; and the third was on social and demographic characteristics of the respondent.

### Population

This study included adults (18 years old or older) from eight European countries: France, Ireland, Italy, the Netherlands, Poland, Portugal, Spain, and Sweden. Based on statistical tables presenting the different sizes of samples according to target population size (*N* > 100,000) — taking a margin of error of 5% and a confidence level of 99% — we concluded that 700 individuals were to be surveyed per country, for a total of 5600 interviews [29]. Since we are expecting a response rate of approximately 30%, samples of 2500 people were drawn for each country.

### Questionnaire

Following a literature review between December 2016, and February 2017 on three electronic databases (MEDLINE, Science Direct, and COCHRANE), we identified 3 main documents on KAP about homelessness [22, 23, 30], with the latter two based on the same instrument. Therefore, the survey questionnaire was designed using these two survey instruments, in addition to ad hoc questions (Tabl**e** 1). Drafts of the questionnaire were discussed within the HOME_EU Consortium study group (see acknowledgment), and the final English version received the approval of all partners.

**Table 1:** Prevalence and KAP items. HOME-EU Opinion Survey. March 2017–December 2017

Measure	Sources	Items
Prevalence	Questions from Tompsett & Toro’s questionnaire (Tompsett et al. 2006 [23]) have been adapted. Questions are preceded by a definition of homelessness as being either roofless or houseless (Edgar et al., 2007 [38]).	Have you ever been homeless? (yes/no) When was It? *(*In the past 12 months/1–2 years ago/3–4 years ago/4–5 years ago/More than 5 years ago/DK /R) How much time in total have you been homeless over your life? (Less than a week/Less than a month/Less than a year/Less than two years/Less than four years/More than four years/DK /R) Has any member of your family, friend or acquaintance ever been homeless? (yes/no/DK /R) Was it …? (Parent [father, mother]/Child/Brother/sister/Husband/wife/ partner/Friend/Other relative/ Acquaintance/DK /R)
Knowledge	Questions from Tompsett & Toro’s questionnaire (Tompsett et al. 2006 [23]) have been adapted.	In your opinion, what is the percentage of people experiencing homelessness with... • Mental disorders? • Addiction problems (alcohol, drugs)?
	Ad-hoc questions	Could you tell me approximately how many people experiencing homelessness there are, in (country)? In (*country*), who funds most social services for homeless people? (Government/ Non-Governmental Organizations -Charities /Churches and religious communities/DK /R) In (*country*), who funds most healthcare facilities for homeless people? (Government/ Non-Governmental Organizations -Charities /Churches and religious communities/DK /R)
Attitudes	Questions are from the Eurobarometer (2010).	In (*country*), in the last 3 years, would you say the number of people experiencing homelessness has …(Strongly increased/Somewhat increased/Somewhat decreased/Strongly decreased/Stayed the same (spontaneous)/DK / R) In your opinion, what are the three reasons that best explain why people become homeless?
	Ad-hoc questions	Should public authorities consider homelessness as a priority? (yes/no/DK /R) In your opinion, who should be mainly responsible for providing… (Government/Non-Governmental Organizations-Charities/Churches and religious communities/ Homeless themselves/ DK /R) • Emergency shelter for homeless people? • Long-term housing for homeless people? Which homeless group should be given priority for a long-term housing program? In general, do you think the Government spends … (Too much/Enough /Too little/DK /R) • On social welfare? • To help homeless people? In your opinion, do services provided...meet the needs of homeless people: (strongly agree /somewhat agree/somewhat disagree/strongly disagree/DK/R) • In hospitals and emergency rooms • By General Practitioners and outpatient specialists • In emergency shelters • In transitional shelters • In the *Housing First* program better meet the needs of people experiencing homelessness when compared to transitional shelter services When passing by a homeless person you are cautious? (Often/Sometimes/Rarely/Never/DK /R)
	Questions from Tompsett & Toro’s questionnaire (Tompsett et al. 2006 [23]) have been adapted.	To reduce homelessness, would you be willing to…>(yes/no/DK /R) • Pay more taxes? • Volunteer? • Have a homeless shelter near your home?
Practices	This question from the Eurobarometer (2010) has been adapted.	In the area where you live, would you say there are (many, some, a few or no) homeless people?
	Questions from Tompsett & Toro’s questionnaire (Tompsett et al. 2006 [23]) have been adapted.	On average, how many different people experiencing homelessness do you see per week? (None/ 1 OR 2/3 TO 10/More than 10/DK/R) Over the past year, have you… (yes/no/DK /R) • Given money, food or clothing to a homeless person? • Given money, food or clothing to a charitable or non-profit organization for homeless people? • Done any volunteer work in a charitable or in a non-profit organization for homeless people?
Opinions regarding core constructs such as: Capabilities Empowerment Community integration Satisfaction with services	Based on the Capabilities approach from Amartya Sen (Sen, 1980 [48]; 1985 [47]; Nussbaum & Sen, 1993; Nussbaum, 2011 [46]).	I will read several statements about homeless people. [Read out item] Please tell me if you… (strongly agree /somewhat agree/somewhat disagree/strongly disagree/DK/R) People experiencing homelessness are the victims of assaults (violence, robbery, threats, and attacks). They are discriminated against in hiring They eat at least two meals a day They are able to keep in touch with family and friends They have a shorter life expectancy than the general population
	Derived from the Empowerment scale by Rogers (Rogers et al. 1997 [36]).	Many remain homeless by choice They could look after (keep clean, decorate) a home if they had one Most have working skills
	Derived from the Community integration approach described by McColl (McColl et al. 2001 [37])	They spend much of their time alone, outside of any social network They have access to paid or unpaid work (volunteering, internship etc.) Their main source of income comes from social welfare benefits

The questionnaire then went through a four-step translation process that included two translations into the targeted native language leading to one consensual version after discussion about discrepancies and input on cultural adaptation provided by each partner within the Consortium. This latter document was translated back into English (back-translation) by two independent professionals, thus producing two back-translations. An expert committee reviewed all the documents mentioned above and the associated reports to produce a final version for the targeted language [31].

A pilot study was conducted on a sample of 30 individuals (mostly French people) to assess the length of the questionnaire and its intelligibility (face validity).

### Measures

#### Sociodemographic characteristics

As social and demographic data have been shown to influence KAP [23, 32] as well as preferences [33], data on socio-demographic characteristics of the respondents were collected, including gender, age, educational level, professional status, annual household income, marital status, number of dependent children, municipality, and the number of adults in the household. In addition, the number of operational landlines and mobile phones within a household was collected to adjust for selectivity due to telephone/mobile ownership.

#### Knowledge, attitudes, and practices towards people experiencing homelessness

##### Knowledge

Knowledge has been defined as any empirical data about homelessness. Five items assessed the respondents’ knowledge of the national prevalence of homelessness and of health issues among people experiencing homelessness — such as mental disorders and addiction issues and the funding of services addressing homelessness (Table 1). The respondents had to provide estimates or select an answer from a set of alternatives.

##### Attitudes

Attitude has been defined as the respondents’ belief or emotional reactions towards people experiencing homelessness, as well as their intention to act to reduce homelessness [34].

Eleven items addressing a respondent’s perception of the capabilities of people experiencing homelessness [35], their empowerment [36], and their integration within the community [37] were created and scored on a four-point scale ranging from 1 = strongly agree to 4 = strongly disagree. Other items drawn from the Eurobarometer 355 on poverty and social exclusion [30] were added to explore a respondent’s perception of the magnitude (1 item) and cause of homelessness (1 item), inclination to help reduce homelessness (3 items), government interventions and spending, including what is and what should be (6 items) (Table 1).

##### Practices

A practice has been defined as any actual past behavior reported by the respondent. Five items were added to gather information about the reported practices of the respondent to reduce homelessness (donations, volunteering), while another item reported on practices to avoid any interaction with people experiencing homelessness (Table 1).

#### Lifetime prevalence of homelessness

Different estimates of homelessness can be produced. Lifetime prevalence of homelessness allows the measurement of the proportion of people within the general population who have ever experienced homelessness at some point during their lifetime. This differs, for example, from a point-prevalence, wherein the proportion of people experiencing homelessness is estimated at a given point in time. One way to obtain an estimate of the lifetime prevalence of homelessness within the general population is to gather the information via a telephone survey [23]. To assess the prevalence of literal homelessness within the lifespan of the respondent and that of his or her relatives and acquaintances, five questions have been included. To avoid any misunderstandings, the definition of homelessness used in this study was given at the beginning of the KAP module as having experienced at least one night of rough sleeping or shelter use. This definition encompasses ETHOS 1 and 2 from the European Typology of Homelessness and Housing Exclusion-ETHOS [38]. Created items are reported in Table 1.

#### Contingent valuation (CV) method

**Fig. 1:**
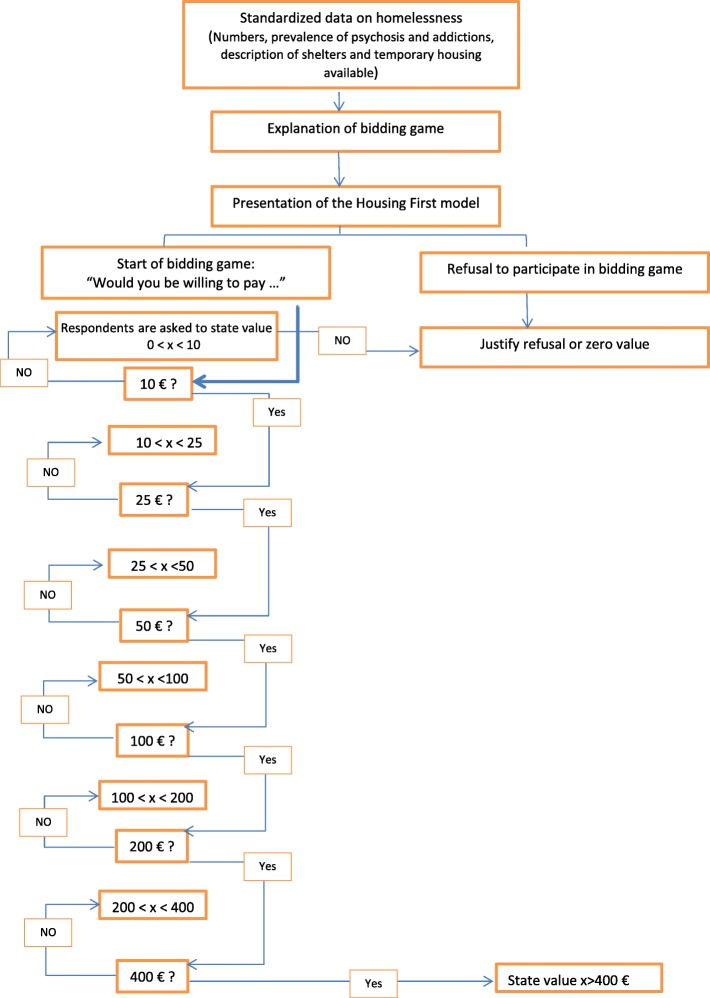
Contingent valuation method with the bidding algorithm

The CV method section was divided into three parts. The first part laid out the CV scenario designed to standardize respondents’ knowledge. This scenario started by presenting information about homelessness, specifically, on the national prevalence of homelessness and on current solutions to accommodate people experiencing homelessness. Then, a description of the HF model was provided, along with data on the proven effectiveness of the HF model drawn from experiments focused on housing stability in several European countries [39]. This description focused on the targeted population and the main characteristics of the HF program such as independent scattered-site supportive housing, low barriers to entrance, having part of the rent paid by the program, and type of team support. The description of the HF model ended with a short presentation of the recent data on the effectiveness of experimental HF programs in the targeted country in terms of housing stability. Thereafter, the elicitation procedure was introduced and explained briefly, followed by information on the bidding process and the payment vehicle, i.e., the means through which the program would be paid for, which is annual general taxation. Finally, the CV scenario ended with a “cheap talk” i.e., a short explanatory passage that is usually employed to mitigate the effects of hypothetical bias by stressing to the respondent the importance of placing realistic bids. Indeed, as respondents are projecting themselves into a hypothetical situation, their bids may be very different from what they would consider paying in reality [40, 41] (Fig. 1).

In the second part of the CV section, since a bidding game format had been chosen to elicit European citizens’ WTP, respondents were presented with increasing bids until they reached an amount they would be unwilling to pay, as follows: *“I will propose amounts in euros. Please tell me what you would be willing to pay each year through taxes for this program. Would you be willing to pay €10?”*. Possible answers were “*yes*”, “*no*”, “*do not know*” and “*refusal to answer*” (Fig. 1). Bids ranged from 10 euros to a maximum of 400 euros; the range had been tested in a small pilot study that included 10 respondents [42, 43]. Bid iterations allow controlling for discrepancies between the respondent’s answer and their future behavior and thus help to reduce hypothetical bias, as answers are more likely to be well-thought-out [26]. To avoid truncated data, a follow-up open-ended question assessed the respondents’ maximum WTP-value by asking them to specify the amount they would be willing to pay between the previously accepted bid and the refused bid [28].

In the third part of the CV section, respondents who refused to take part in the bidding process or those who spontaneously gave a WTP-value of zero were asked additional close-ended and open-ended questions to categorize their answers as *genuine zeros* or *protest-zeros*. Possible responses were: *“The program won’t work”; “Other programs are more important/of higher priority”*; *“I cannot afford to pay more taxes”; “I do not want to pay more taxes”*; “*Other (please specify)*”. Participants who answered with one of the first three answers will be categorized as genuine zeros, as they either did not value the HF model or lacked funds [44].

Other responses will be categorized as protest zeros, as respondents either contested the payment vehicle — taxes — or even the survey instrument. Finally, following preference elicitation, respondents were asked to weigh the certainty of their answers on a four-point scale ranging from 1 “absolutely sure” to 4 “absolutely unsure”.

### Data analysis

#### Descriptive analysis

Sociodemographic data, prevalence estimates of homelessness, KAP answers, and WTP estimates will be presented descriptively as the means, medians, or proportions and will be stratified by gender and country. Parametric and non-parametric statistics will be used to compare the knowledge, attitudes, practices, and maximum WTP differences between countries, respondent profiles and other relevant subgroups. Data collected in the questionnaire will be used to create several KAP indicators such as: “Good knowledge”, “Poor knowledge”, “Attitude of contest”, “Attitude of no contest”, “Exposed practice”, and “unexposed practice”.

Evidence suggests that attitudes about homelessness are influenced by one’s experience and perceptions regarding social justice, social choice and unfairness [45]. Therefore, we will create a composite indicator translating European’s level of tolerance about homelessness (latent trait). To build this indicator, we will use different measures assessing the perception participants may have about people experiencing homelessness through the previously mentioned items on capabilities [35, 46–48], empowerment [36], and community integration [37]. In addition, we will use questions targeting attitudes about public policy.

The psychometric properties of these indicators will be assessed: the construct validity will be ascertained using principal component analysis, and unidimensionality will be assessed using a Rasch analysis. The goodness-of-fit statistics (INFIT, ranging between 0.7 and 1.3) will ensure that all items of the scale measure the same concept. Reliability will be assessed using Cronbach’s alpha coefficient [49].

#### Predictive analysis

Dichotomous and score variables will be analyzed using generalized estimating equations (GEE – GENLIN function) with binomial or normal linear distribution, respectively, and a set of independent variables (sociodemographic data, knowledge about and experience with homelessness). The within-subject “random” effects will be modeled for each participant to adjust standard errors for the non-independence of observations within participants (cluster parameter = ‘countries’; *n* = 8).

An important consideration for the multivariate analyses of the WTP is the level of reported zeros [50, 51]. To take into account the existence of “protest zeros”, a zero-inflated negative binomial (ZINB) model will be tested [52–54]. The ZINB is a two-part model consisting of a logit model and a negative binomial model. The logit component will be used to model the predictors of protest behaviors by modeling the factors associated with zero values — both genuine and protest zeros. The predictors of the valuation of the HF model will be assessed through the negative binomial part of the model. The relevance of using a ZINB regression will be confirmed by calculating the Vuong test [55].

Descriptive analyses and econometric analyses will be performed using the computer software SPSS 12 for Windows (SPSS Inc., IBM, NY, USA), as well as RStudio version 3.2.1 software (RStudio, Inc., Massachusetts, USA).

## Discussion

In Europe, hardly any studies have explored the views and attitudes of the general population about homelessness and programs targeting its reduction. Thus, this study will address the gap in knowledge about Europeans’ views of homelessness by conducting a knowledge, attitudes, and practices (KAP) survey in eight European member states; similar studies have mostly been conducted in the United States of America [22, 23]. It will also be an opportunity to provide updated data on the lifetime prevalence of literal homelessness among the general population of Italy [24] and expand the scope of countries to include seven other European countries. In addition, this study will be the first to provide valuation data for the Housing-First (HF) model through a contingent valuation (CV) method.

HF has been experimented with in North America and in Europe and has provided evidence of housing stability and of healthcare cost reduction [21] for long-term homeless people with severe mental illness. This telephone survey took place in countries where established HF programs have been presented as showing the most fidelity to the original Pathways to Housing First program (i.e., France, Ireland, Portugal and the Netherlands) [39]. Additionally, included in this study are Italy and Spain, where HF programs are fairly recent [56–58]; Sweden, where HF programs, though long-standing, present the most variations to the PHF approach [59]; and Poland, where no Housing First initiatives exist to date.

In this study, we collected data via telephone both for the KAP survey and the CV method. While internet surveys provide flexibility to respondents in terms of time and place of completion, they tend to exclude people from areas with incomplete coverage, elderly people and low-income households [60]. Additionally, as one of our purposes is to explore the value Europeans place in the Housing First model, the CV method was preferred over choice-based techniques like discrete-choice experiments [61]. The latter would require visual support to present several adaptations of the HF model, which would differ only by varying service levels or a set of characteristics. In addition, a telephone survey is the closest option to a face-to-face interview and the most recommended alternative for preference elicitation [44].

However, some limitations have to be mentioned. First, since homelessness is a sensitive topic, respondents may have felt uncomfortable to voice their true opinion, making social desirability bias a potential issue. However, anonymous telephone surveys usually allow more self-expression than face-to-face interviews. Additionally, interviewers were trained not to skew answers. Second, possible selection bias of the study population is associated with quota sampling, with a tendency to underrepresent people difficult to interview or contact [62]. Additionally, telephone surveys are often associated with the under-sampling of younger respondents [63]. However, to properly represent this group, interviewers were instructed to callback fifteen times before discarding a landline or cell phone number and offer appointments to either start or complete an interview. Moreover, decreasing willingness to participate in telephone surveys [64] may give rise to nonresponse bias; statistical methods such as weighting or a regression-based model will be used if the data need correction for the lack of representativity [65]. Likewise, should missing data call for statistical handling, following the assumptions about their underlying mechanism, appropriate imputation methods will be implemented [66].

Third, it is likely that lifetime prevalence may be underestimated due to the exclusion of individuals who cannot afford a telephone but also of those who are currently experiencing homelessness. Fourth, excess zeros can be expected in the CV method for, mainly, two reasons. One, the payment vehicle chosen i.e., annual taxation may have deterred participation or lead respondents to place a null value on the model — as evidenced by a recent study in which zero bids represented half of the total bids [27]. This choice was justified, as the health care system in the targeted European countries is mostly funded through general taxation. Two, the respondents were asked to state their willingness to pay for a program they are likely to never benefit from. An American study found that respondents are more willing to pay for programs that will benefit themselves or their relatives [33]. Even considering the difference in the social contract between the USA and Europe, excess zeros can be expected in this study, as people experiencing homelessness are socially marginalized. Anticipating the excess zeros, data analysis will be carried out using appropriate statistical models such as the zero-inflated model [53].

### Potential impact

The results from the KAP survey will make known Europeans’ views of homelessness, especially misconceptions that could be barriers to the implementation of future programs. Ultimately, as the number of people experiencing homelessness is increasing all over Europe, the CV results could be instrumental for key stakeholders in understanding the level of support from the general population for programs such as Housing First, especially considering that the determinants of this valuation will also be examined according to the social and political context of each country.

## Abbreviations

CATI: Computer Assisted Telephone Interviews
CV: Contingent Valuation
ETHOS: European Typology of Homelessness and Housing Exclusion
GEE: Generalized Estimating Equations
GEE-GENLIN: GEE models with the SPSS software using the GENLIN command.
HF: Housing First
INFIT: Information-weighted Fit
KAP: Knowledge, Attitudes, and Practices
USA: United States of America
WTP: Willingness-To-Pay
ZINB: Zero-Inflated Negative Binomial model
